# Neuropathy in Val122Ile Hereditary Transthyretin (ATTR) Amyloidosis: A Multicenter Retrospective Cohort Study

**DOI:** 10.1111/jns.70160

**Published:** 2026-08-28

**Authors:** Anna Paula Paranhos, Marcela Câmara Machado Costa, Carolina Lavigne Moreira, Diogo Fernandes dos Santos, Marcondes Cavalcante França, Renan Flavio de França Nunes, Osvaldo José Moreira do Nascimento, Jemima Araujo da Silva Batista, Eduardo Boiteux Uchôa Cavalcanti, Cleonisio Leite Rodrigues, Ândrea Virgínia Ferreira Chaves, Marcelo Soares Kerstenetzky, Lara Albuquerque de Brito, Carlo Domênico Marrone, Marcus Vinicius Magno Gonçalves, Pedro José Tomaselli, Wilson Marques

**Affiliations:** ^1^ Hospital das Clínicas Federal University of Pernambuco Recife Pernambuco Brazil; ^2^ RARUS Reference Service for Rare Diseases in Pernambuco Recife Pernambuco Brazil; ^3^ Faculty of Medicine of Ribeirão Preto University of São Paulo Ribeirão Preto São Paulo Brazil; ^4^ Escola Bahiana de Medicina e Saúde Pública Salvador Bahia Brazil; ^5^ Faculty of Medicine Federal University of Uberlandia Uberlândia Minas Gerais Brazil; ^6^ Faculty of Medicine UNICAMP Campinas São Paulo Brazil; ^7^ Universidade Federal Fluminense Niterói Rio de Janeiro Brazil; ^8^ Universidade Federal do Vale do São Francisco Petrolina Pernambuco Brazil; ^9^ Rede Sarah—Serviço de Reabilitação Neurológica Brasília Distrito Federal Brazil; ^10^ Hospital Geral de Fortaleza Fortaleza Ceará Brazil; ^11^ Hospital São Lucas da PUCRS Porto Alegre Rio Grande do Sul Brazil; ^12^ Instituto Nacional de Ciência e Tecnologia em Saúde Mental Digital Porto Alegre Brazil

**Keywords:** amyloidosis, ATTRv, neuropathy, TTR, Val122Ile

## Abstract

**Background and Aims:**

The Val122Ile ATTR Amyloidosis has traditionally been linked to cardiac manifestations. Recent studies suggest that neuropathy may be relevant. In this study, we characterized its peripheral nerve manifestations in depth.

**Methods:**

This was a national, multicenter, observational, retrospective study. Patients underwent careful clinical and neurophysiological evaluation. We excluded those with alternative etiologies.

**Results:**

We identified 246 Val122Ile carriers, including 240 heterozygous, 4 homozygous, and 2 compound heterozygous. Gender distribution was similar. Age of onset: 19–89 years, mean 53 ± 17. Self‐reported ethnicity: White (26.7%), Black (17.3%), and Mixed (Pardo) (56.0%). Birthplaces spanned all Brazilian regions, mainly the Northeast. Among heterozygotes, 52 of 122 (42.6%) were symptomatic. Carpal tunnel syndrome (CTS) preceded polyneuropathy and/or cardiomyopathy in 69.2%. The most frequent onset manifestations, excluding CTS, were cardiomyopathy (52.9%), neuropathy (37.3%), and mixed phenotype (9.8%). Age of onset: 29–86 years (mean: 64.9 years). During follow‐up, 14/27 cardiac patients developed neuropathy, and 8/19 neurologic patients developed cardiomyopathy, significantly increasing the mixed phenotype from 9.8% to 52.9%. Most patients with neuropathy presented with sensory or sensory and motor disease (62.2%), followed by small fiber neuropathy (32.4%), and isolated dysautonomia (5.4%).

**Interpretation:**

Neuropathy is underrecognized in Val122Ile ATTR Amyloidosis, is frequent at disease onset, may be the sole manifestation, and may occur in White persons. CTS frequently preceded neuropathy, usually an axonal polyneuropathy, although atypical patterns were observed. This variant clusters in the Northeast region of Brazil.

## 1 Introduction

Hereditary Transthyretin (ATTRv) Amyloidosis is a rare, autosomal dominant inherited disease resulting from pathogenic variants in transthyretin (*TTR*) gene [1]. More than 140 *TTR* gene variants are associated with the disease. It is a systemic disease that primarily affects the heart and the peripheral nervous system, and its clinical presentation is partially determined by genotype–phenotype correlations [1].

The prevalence of the *TTR* Val122Ile allele varies significantly according to ethnic background, presumably affecting 3%–4% of the Afro‐Americans [2], 0.02% of Caucasians [3], and 1.3% of those with Hispanic/Latino ancestry [4]. The Val122Ile founder variant appears to have originated in West Africa [4], where its frequency is high [5]. A significant proportion of the Brazilian Black population, particularly those in the Northeastern region, trace ancestry to West Africa [6].

Despite its high prevalence [7], the penetrance of *TTR* Val122Ile is incomplete and low, and most carriers do not manifest the disease [3]. As expected, this variant has already been reported in Brazil [8, 9], a country whose population is highly miscegenated, resulting mainly from a historical mixture of Europeans (mostly from Portugal, but also from Italy, Spain, Germany, Poland, the Netherlands, France, among other European countries), Africans and Indigenous peoples [10]. African ancestry is present all over Brazil, but it is mostly prevalent in the Northeastern region [10].

This variant is characteristically associated with a late‐onset hypertrophic restrictive cardiomyopathy [4], which is frequently preceded by carpal tunnel syndrome (CTS) [11, 12]. Interestingly, patients carrying homozygous variants seem to have an early‐onset cardiac disease [13]. Spinal stenosis [14], and polyneuropathy have also been described [15, 16], although the polyneuropathy is usually considered minor in this genotype [17, 18].

Recently, however, this concept has been challenged. A small Italian series of Caucasian patients carrying the *TTR* Val122Ile variant showed the presence of an axonal sensorimotor polyneuropathy in four out of nine symptomatic patients [15]. In addition, a phenome‐wide association study scanning International Classification of Diseases (*ICD*) diagnosis codes in biobank participants of African ancestry demonstrated that polyneuropathy prevalence among *TTR* Val122Ile carriers ranges from 2.1% to 9.0%. These findings show that the Val122Ile carriers are at increased risk of developing polyneuropathy [11]. In a United Kingdom–based cohort of 52 individuals with this variant, neuropathy was identified in approximately 40% of cases [16].

It seems clear now that concepts about the consequences of Val122Ile are changing [11, 14, 15, 16]. It is essential to better characterize its prevalence, clinical aspects, and neurophysiological findings. Our study aims to evaluate the presence of neuropathy in Brazilian patients with this variant and to determine its demographic, clinical, and neurophysiological characteristics.

## 2 Materials and Methods

A national, multicenter, observational, retrospective cohort study was conducted between December 2021 and April 2023. Demographic, clinical, and neurophysiological data were collected using a standardized pro forma. Individuals aged 18 years or older with the confirmed *TTR* Val122Ile variant from 10 referral centers for neuromuscular disorders across Brazil were included. Data were cross‐checked to prevent duplicate entries.

All individuals underwent a demographic assessment. Patients were selected for analysis of clinical and neurophysiological features using the following exclusion criteria: compound heterozygosity in the *TTR* gene, comorbidities or other factors known to predispose to neuropathy (Table S1), or patients who had not yet undergone neurophysiological studies (relatives of index patients identified through family screening). Other causes of neuropathy were evaluated and ruled out, including confirmed metabolic conditions (diabetes mellitus), malnutrition, chronic alcoholism, and autoimmune disorders (systemic lupus erythematosus, rheumatoid arthritis, Sjogren's disease), motor neuron disease, exposure to toxic drugs (Tacrolimus and Amiodarone), infectious diseases (leprosy, HIV, hepatitis and Lyme's disease), vitamin deficiencies (vitamin B12 or folic acid), inflammatory conditions such as chronic inflammatory demyelinating polyradiculoneuropathy and patients with other hereditary neuropathies.

For clinical and neurophysiological analysis, patients were grouped into symptomatic and asymptomatic categories. For the purposes of this study, we assumed the following definitions in relation to peripheral nerve involvement: (a) Asymptomatic carriers: no sensory, autonomic, or motor complaints; normal neurologic examination; normal nerve conduction studies (NCS)—not CTS; normal needle electromyography (EMG); normal sympathetic skin responses; (b) Polyneuropathy: presence of length‐dependent manifestations; (c) Sensory polyneuropathy: presence of sensory complaints, sensory abnormalities on neurologic examination, and abnormal sensory NCS; (d) Sensory and motor polyneuropathy: additional presence of motor complaints; presence of weakness and atrophy on neurologic examination; abnormal sensory and motor nerve conduction studies and needle EMG; (e) Small fiber neuropathy (SFN): length‐dependent (or distal) presence of pain or other positive sensory complaints and thermoalgesic sensory abnormalities on neurologic examination, with normal sensory and motor NCS. Dysautonomia could be present; (f) Pure dysautonomia manifestations: none of the abnormalities previously described and presence of any of the following manifestations: orthostatic intolerance/hypotension with no variability in cardiac frequency; fecal or urinary retention or incontinence; incomplete bladder emptying; persistent diarrhea, early satiety, impotence, unintentional weight loss, trouble with light accommodation. CTS was considered a prodromal manifestation and therefore excluded from the definition of disease onset.

Cardiac involvement was defined as the presence of an interventricular septum > 11 mm, a positive 99mTc‐pyrophosphate (PYP) cardiac scintigraphy, or a positive endomyocardial biopsy.

Patients with biallelic Val122Ile variants were analyzed separately and compared with those with heterozygous variants. Since this was a retrospective and multicenter study, not all patient data were available for analysis. The study was approved by the local Ethics Committee at each site. All participants provided written informed consent.

### 2.1 Statistical Analysis

Descriptive statistics are presented as means (standard deviations) and medians for continuous variables and as percentages (frequencies and distributions) for categorical variables. A Student's *t*‐test was used to compare means between groups. A *p*‐value of < 0.05 was considered significant.

## 3 Results

### 3.1 Demographic Aspects

A total of 246 individuals from 84 kindreds with the Val122Ile variant in the TTR gene were identified. Of these, 240 were heterozygous, four were homozygous, and two were compound heterozygous for a variant in the *TTR* gene (Val122Ile/Val30Met and Val122Ile/Ile107Val). There was an equal distribution of males and females. Age ranged from 19 to 89 years, with a mean of 53.4 years (±17.3) and a median of 51.0 years (40.0–68.5). Race was self‐reported as: 56.0% mixed (Black/White—Pardo), 26.7% White and 17.3% Black.

**FIGURE 1:**
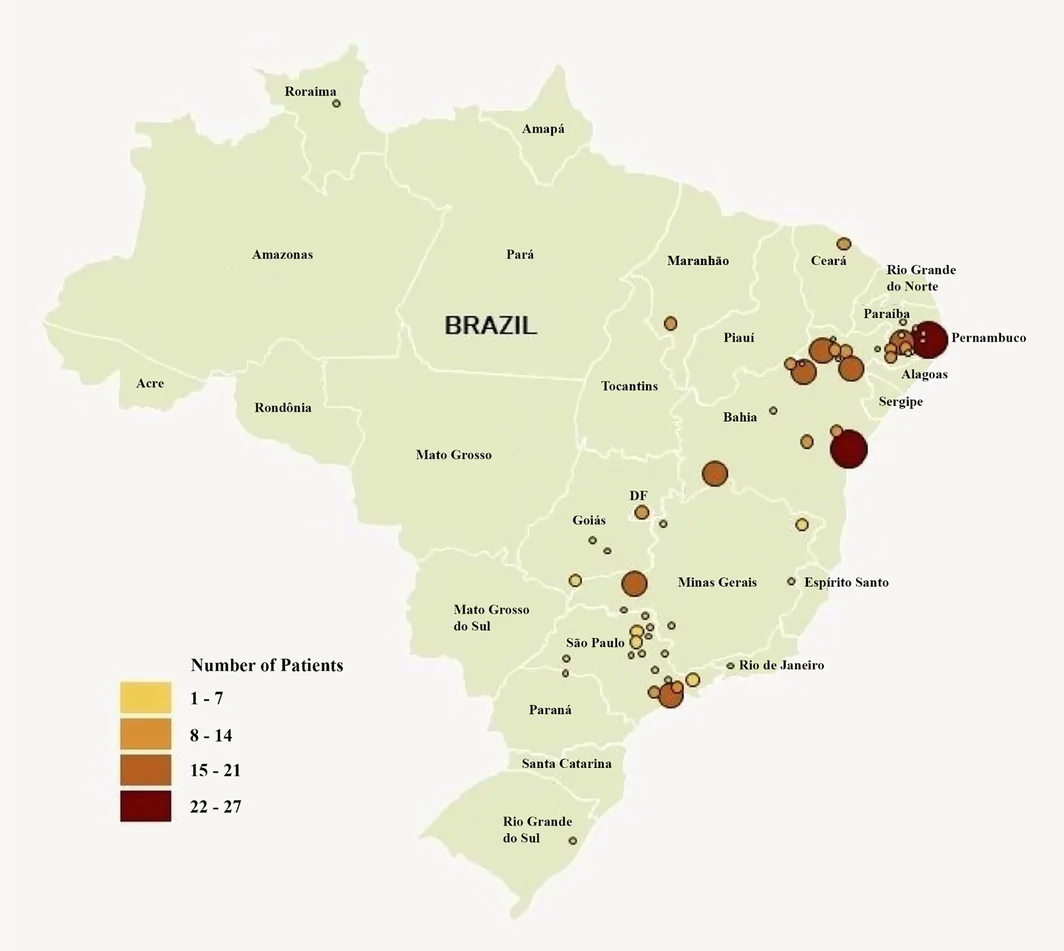
Map with frequency distribution of the birthplace of individuals with *TTR* Val122Ile variant in Brazil.

Individuals were born in all regions of Brazil, mainly in the states of Pernambuco (32.4%), Bahia (32.0%), São Paulo (16.4%), and Minas Gerais (8.6%). A greater concentration was observed in the cities of Salvador (11%), Recife (8.9%), Uberlandia (4.5%), and cities along the banks of the São Francisco River, on the border of the states of Pernambuco and Bahia (Figure 1). Regarding the state of residence, the distribution was also maintained across all regions of the country, mainly in the same states: Pernambuco (32.0%), Bahia (29.5%), São Paulo (22.5%), and Minas Gerais (4.9%).

### 3.2 Clinical and Neurophysiological Aspects

**FIGURE 2:**
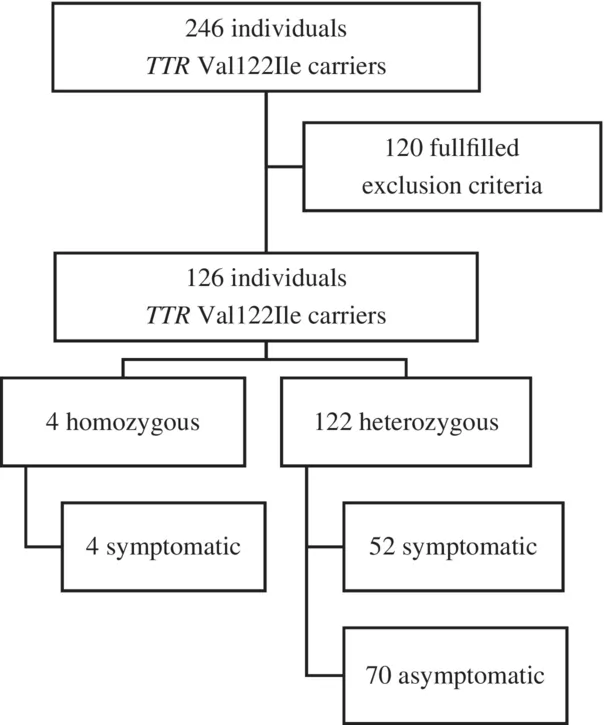
Study sample population flow diagram.

Of the 246 individuals with the *TTR* Val122Ile variant, 120 met one or more of the exclusion criteria (Table S1) for the clinical and electrophysiological analysis (Figure 2).

Among the 126 individuals analyzed, 122 were heterozygous and four were homozygous for the Val122Ile variant. Of the 122 heterozygous individuals, 52 (42.6%) were symptomatic. While the whole group exhibited a slight predominance of females (54.1%), the symptomatic group showed a mild male predominance (55.8%). The symptomatic group was significantly older than the asymptomatic carriers (ranging from 29 to 86, a mean of 68.9 ± 12.4 years vs. 20 to 69 years, 42.4 ± 11.2 years, *p* < 0.001) (Table 1).

**TABLE 1:** Summary of the demographic and clinical characteristics of individuals with the *TTR* Val122Ile variant.

Variable	*N* ^a^	
Number of carriers	246	
Females/males		124/122
Age in years (range, mean ± SD) *N* = 228^b^		19–89; 53.4 ± 17.3
Race/ethnicity^c^	243	
White		65 (26.7%)
Black		42 (17.3%)
Mixed (Black/White)		136 (56.0%)
*TTR* Val122Ile variant	246	
Heterozygous		240 (97.6%)
Compound heterozygous		2 (0.8%)
Homozygous		4 (1.6%)
Heterozygous carriers included in the clinical analysis	122	
Females/males		66/56
Age in years (range, mean ± SD)—*N* = 116^d^		20–89; 52.9 ± 17.4
Asymptomatic	70	
Females/males		43/27
Age in years (range, mean ± SD) *N* = 70		20–69; 42.4 ± 11.2
Symptomatic	52	
Females/males		23/29
Age in years (range, mean ± SD) *N* = 46^d^		29–89; 68.9 ± 12.4
CTS as a previous event		36 (69.2%)
Age at onset of CTS manifestations (range, mean ± SD)		34–87; 56.5 ± 16.1
Age at onset of manifestation (not CTS) (range, mean ± SD)		29–86; 64.9 ± 13.4
Main manifestation at onset	51^e^	
Cardiomyopathy		27 (52.9%)
Neuropathy		19 (37.3%)
Mixt phenotype		5 (9.8%)
Cumulative involvement	51^f^	
Cardiomyopathy		13 (25.5%)
Neuropathy		11 (21.6%)
Mixt phenotype		27 (52.9%)
Coutinho stages of polyneuropathy	39	
Stage 1		36 (92.3%)
Stage 2		3 (7.7%)
Stage 3		0
Neuropathy	37^g^	
Sensory or sensory‐motor neuropathy		23 (62.2%)
Small fibers neuropathy		12 (32.4%)
Isolated dysautonomia		2 (5.4%)

^a^ *N* in the second column of the table refers to the number of patients with data available for analysis.

^b^ The age data from 7 patients were not available and the age of 11 patients who had died were not analyzed.

^c^ Data from 3 participants regarding race/ethnicity were not available.

^d^ The age data from 1 patient was not available and the age of 5 patients who had died were not analyzed.

^e^ Data from 1 patient were not available regarding the onset of clinical manifestations.

^f^ Cardiological investigation at follow‐up was not available in 1 patient with no cardiologic symptoms.

^g^ Data were not available regarding classification of polyneuropathy for 2 patients.

The mean time to diagnosis among index patients was 3.6 ± 2.9 years, ranging from 1 to 12 years. CTS symptoms preceded disease onset by an average of 8.4 years in 69.2% of patients. The age at disease onset, excluding CTS symptoms, ranged from 29 to 86 years, with a mean of 64.9 ± 13.4 years. In the group of homozygous patients, disease onset ranged from 60 to 63 years, with a mean of 61.7 ± 1.5 years, but the difference was not statistically significant (*p* = 0.72).

**FIGURE 3:**
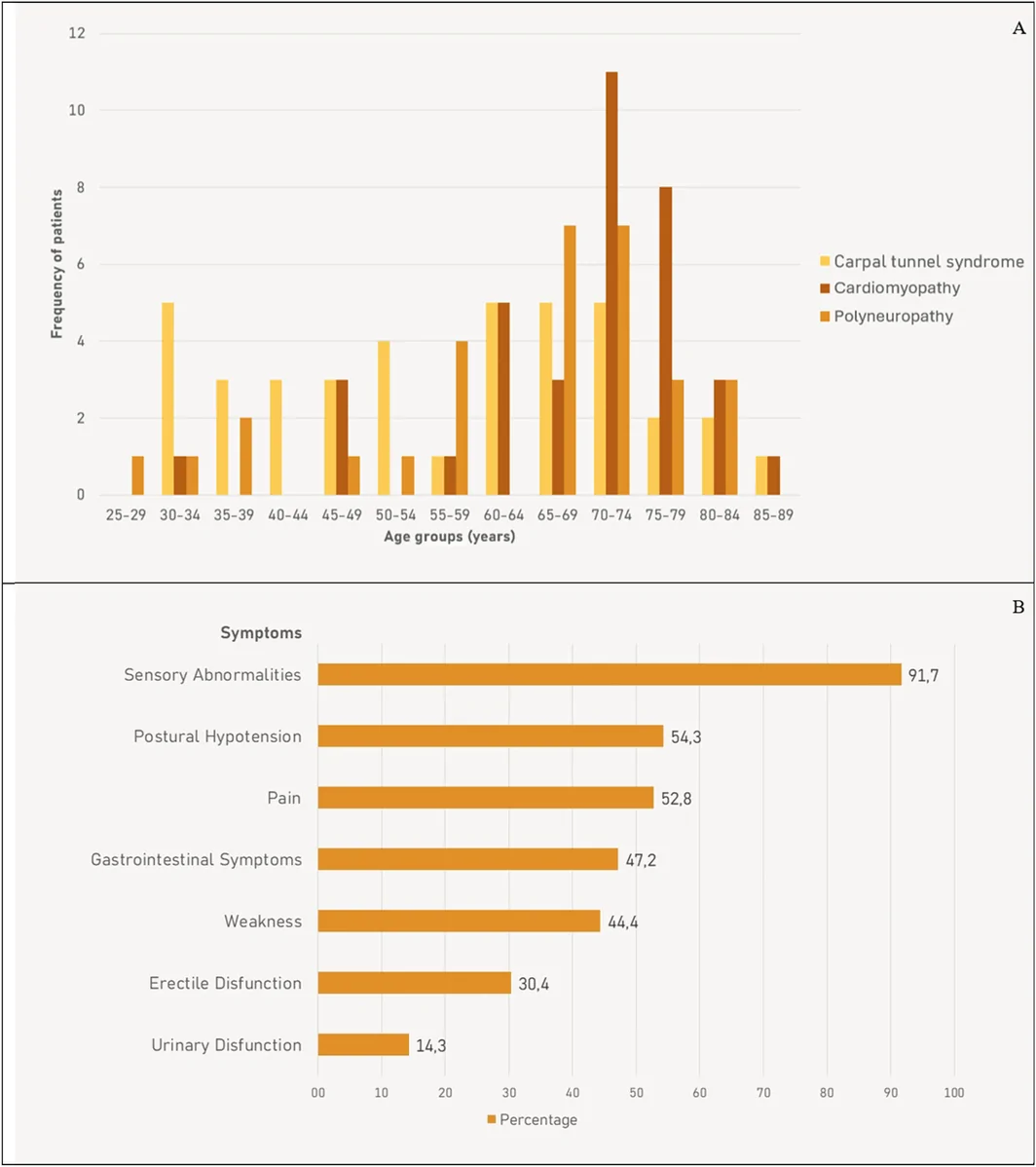
(A) Carpal tunnel syndrome, cardiomyopathy, and polyneuropathy manifestations distributed according to age of manifestation onset in patients with ATTR Val122Ile Amyloidosis. (B) Distribution of symptoms presented by patients with polyneuropathy.

In the heterozygous symptomatic patients, the mean age at onset was 68.6 years (±11.6 years) for cardiomyopathy, 63.9 years (±14.6 years) for neuropathy, and 56.5 years (±16.1 years) for CTS (Figure 3A). During the follow‐up period (ranging from one to 12 years, mean of 4.7 years), 14 (51.9%) of the 27 patients whose disease started as cardiomyopathy developed neuropathy. On the other hand, among the 19 patients who presented neuropathy as the first manifestation, eight (42.1%) developed cardiomyopathy. The mixed phenotype subsequently increased from five to 27 (52.9%) patients.

Of the 122 heterozygous individuals who underwent NCS/EMG, 61 showed abnormalities on the exam, with some having more than one change. The findings in symptomatic and asymptomatic patients are shown in Table 2. Among patients with non‐focal neuropathies, the most frequent findings were sensory and motor axonal polyneuropathy (18/37, 48.7%), followed by sensory axonal polyneuropathy (4/37, 10.8%). Uncommon, generalized findings included multiple mononeuropathy and CIDP‐like patterns. Twelve of the patients with non‐focal neuropathies had a normal NCS/EMG examination.

**TABLE 2:** NCS/EMG findings in symptomatic and asymptomatic patients with ATTR Val122Ile amyloidosis.

NCS/EMG findings^a^	*N* (%)
Asymptomatic patients	70
Carpal tunnel syndrome	14/70 (20.0%)
Radiculopathy	2/70 (2.9%)
Normal NCS/EMG	56/70 (80%)
Symptomatic patients	52
Focal neuropathy and/or radiculopathy	
Carpal tunnel syndrome	36/52 (69.2%)
Cubital tunnel syndrome	6/52 (11.5%)
Radiculopathy	6/52 (11.5%)
Non‐focal neuropathy	37^c^
Sensory axonal polyneuropathy	4/37 (10.8%)
Sensory‐motor axonal polyneuropathy	18/37 (48.7%)
CIDP‐like^b^	1/37 (2.7%)
Multiple mononeuropathy	2/37 (5.4%)
Normal NCS/EMG	12/37 (32.4%)

^a^ NCS/EMG: nerve conduction studies (NCS) and electromyography (EMG).

^b^ CIDP‐like: chronic inflammatory demyelinating polyradiculoneuropathy‐like fulfilling the EAN/PNS 2021 criteria [19].

^c^ Data were not available regarding classification of non‐focal neuropathy for 2 patients.

In addition to the generalized findings, CTS (36/52, 69.2%) and ulnar nerve cubital syndrome (6/52, 11.5%) were frequent focal findings. Considering the whole group (symptomatic and asymptomatic patients), CTS when present was bilateral in 95.8% of the cases. Radiculopathy was found in 6/52 (11.5%) of the symptomatic patients.

SSR evaluation was performed in 92 patients, and SSR was absent in 16 (17.6%) of the 91 patients with available examination data. The absence of SSR as an isolated neurophysiological change was not observed in any of the patients: nine presented polyneuropathy (in six patients associated with CTS), one presented multiple mononeuropathy (plus CTS), and six presented CTS and/or cubital tunnel syndromes. In terms of the neuropathy pattern, SSR was absent in eight (57.1%) of the 14 patients with polyneuropathy and in the only patient with multiple mononeuropathy who underwent SSR testing. SSR was normal in the patient presenting with a CIDP (chronic inflammatory demyelinating polyneuropathy)‐like phenotype.

Finally, based on the clinical evaluation and NCS/EMG patterns, we classified patients with neuropathy into 5 groups: sensory polyneuropathy/sensory‐motor polyneuropathy, multiple mononeuropathy, CIDP‐like patterns, small fiber neuropathy, and isolated dysautonomia, with definitions provided in the Methods section. Sensory manifestations were the most common abnormality, observed in 91.7% of patients with neuropathy. Weakness was reported by 44.4% of patients. Symptoms associated with dysautonomia such as postural hypotension, gastrointestinal symptoms (diarrhea, constipation, early satiety, fecal incontinence), and urinary dysfunction (incontinence, incomplete voiding) were observed in 54.3%, 47.2%, and 14.3%, respectively, while erectile dysfunction was found in 30.4% of males. Approximately half of the patients reported neuropathic pain (Figure 3B). Among patients with neuropathy with large‐fiber impairment (sensory or sensory‐motor polyneuropathy), clinical and/or neurophysiological data indicated that 75.0% also had small‐fibers impairment. All patients with polyneuropathy were treated with tafamidis at 20 mg/day, as it was the only disease‐modifying therapy available through the Brazilian public healthcare system (Sistema Único de Saúde, SUS), with only the 20 mg/day dose formulation available during the study period.

## 4 Discussion

This multicenter study, which encompassed all Brazilian regions, has enabled a better understanding of the scenario involving Val122Ile ATTR amyloidosis. The Val122Ile variant was identified in a significant number of individuals and families. It was found in Black, Pardo and White populations, with no representation of other ethnicities. Contrary to general expectation, and according to self‐declared origin, White individuals outnumbered Black individuals (26.7% vs. 17.3%), although the majority (56.0%) were Pardo, thus supporting Brazil as a highly miscegenated country.

The *TTR* Val122Ile variant has been widely described in the Black population (African and Afro‐American descendants) [2, 3, 4, 5, 7]. Recently, however, it was described in a small Italian cohort of Caucasian patients [15]. The origin of this variant is uncertain.

In our study, race was self‐declared, and certainly many of the self‐declared White people carry African genes and possibly genes of other origins, like the original Indian population, Japanese, among other populations, as the country received immigrants from different regions of the world [10]. Unfortunately, it was not possible to use molecular markers of ancestry in this study.

The Val122Ile variant shows a high concentration in the Northeastern region, an endemic area, particularly in the states of Bahia and Pernambuco, which can be explained by a possible common immigration effect. In these states, there is a significant population of African descent, comprising a large number of Black and Pardo people [10]. Supporting this endemicity, we identified four homozygous patients in the Northeast region, who belong to families with a history of consanguinity, living in small villages. In addition, we also found two compound heterozygous patients (Val122Ile/Val30Met and Val122Ile/Ile107Val) in the same area, suggesting that the endemicity may also involve other variants. The other states with high concentration, Sao Paulo and Minas Gerais, received in the past a huge number of immigrants from the Northeast region.

Regrettably, Brazil received approximately 5 million enslaved from Africa between 1500 and 1850, primarily from two regions: West Africa and Central Africa [6]. The Brazilian endemic areas are closely associated with the West African slave route (Figure S1), coinciding with genetic analyses that reveal a high prevalence of the Val122Ile allele in this region [5]. The diverse ethnic origins of Brazil's Black population likely explain the higher prevalence of this variant in the states of Bahia and Pernambuco (Northeast Brazil). Our study observed a high concentration of individuals with the Val122Ile variant in the state capitals (Recife and Salvador) and in cities along the banks of the São Francisco River, on the border of the states of Pernambuco and Bahia. This region is home to numerous quilombos, communities originally formed by enslaved Africans who resisted the slavery regime, where their descendants continue to live today [10].

There was a slight male predominance among patients in the symptomatic group. This may suggest biological factors that inhibit amyloid deposition in females or facilitate it in males with the Val122Ile variant [20]. Recent studies have demonstrated that myocardial involvement is more frequent and pronounced in male patients with ATTRv amyloidosis [20]. Certain variants exhibit sex‐specific penetrance and phenotypic variation. Data from the THAOS survey have shown that the Val122Ile variant has greater male prevalence [20].

The mean time to diagnosis in index patients was 3.6 years. Diagnosis is often delayed because disease manifestations can vary and be nonspecific [1]. Although the age of symptom onset varied widely, most patients experienced late‐onset disease (after age 50). Only 11.8% of patients had an early onset: one patient presented with cardiologic symptoms at age 45, and five patients presented with early‐onset polyneuropathy, the youngest at age 29. All patients in the homozygous group experienced late‐onset disease; however, their onset was, on average, 2.1 years earlier than that of heterozygous patients, consistent with evidence reported in the African American population [13]. However, the number of homozygous patients in our study was small, preventing a definitive comparison.

In most patients, CTS symptoms preceded the onset of the disease, defined by the presence of neuropathy and/or cardiomyopathy, by an average of 8.4 years. This should serve as a warning sign for Val122Ile carriers. Although the *TTR* Val122Ile variant is classically associated with cardiomyopathy, neuropathy is frequent at disease onset and is the only manifestation in 21.6% of patients after a median follow‐up of almost 5 years. It was also notable that the mean age at onset of neuropathy symptoms was 4.7 years earlier than that for cardiomyopathy. However, there may have been selection bias, with patients with neuropathy referred earlier to neurological referral centers. Consistent with our findings in the Val122Ile variant, patients with the Glu89Gln usually manifest a mixed phenotype, and neuropathy precedes cardiac disease for a prolonged period [21].

In the present study, isolated cardiomyopathy was the presenting manifestation in more than half of patients, whereas a mixed phenotype (cardiomyopathy and polyneuropathy) was the most common clinical presentation in a recent North American study [14]. In a United Kingdom cohort, a lower frequency of neuropathy was reported in patients with Val122Ile ATTR amyloidosis, suggesting phenotypic variability associated with this variant across different countries [16]. Although the cause of these discrepancies has not been specifically studied, it is possible that the availability of more advanced diagnostic protocols, such as skin biopsy and sweat gland nerve fiber density tests, may account for these differences [14].

Among patients with neuropathy, sensory symptoms were the most common. Unlike ATTR Val30Met amyloidosis [1], a lower frequency of dysautonomic symptoms and neuropathic pain was reported, suggesting less frequent involvement of the small nerve fibers. Motor impairment is also less frequent and, when present, milder than that seen in Val30Met patients [1]. Similar findings were reported in a recent study that retrospectively analyzed data from 31 patients with ATTR Val122Ile amyloidosis [14].

Among carriers of *TTR* Val122Ile variant, half (61 out of 122) showed abnormal NCS/EMG results, including clinically asymptomatic carriers. Most patients presented the classic described patterns, although atypical patterns were also observed. CTS was the most frequent finding, typically almost always bilateral, as reported by other authors [14, 22]. Although CTS is the most frequent focal peripheral neuropathy in the general population with a lifetime prevalence estimated at 3.1% [23], it was present in 69.2% of symptomatic patients and 20.0% of asymptomatic carriers, thus demonstrating a remarkable difference.

Cubital tunnel syndrome, an injury of the ulnar nerve at the elbow, was found in 11.5% of the symptomatic patients. This number is likely underestimated, since some centers do not include the elbow region in their routine nerve conduction protocol. To the best of our knowledge, there has been only one previous report of this syndrome in a patient with the homozygous Val122Ile variant [24]. Apparently, no studies have reported the frequency of this finding in patients with ATTRv amyloidosis. We suggest the importance of further studies, as this may be a limiting entrapment neuropathy [25], found in 34% of patients with a diagnosis of wild‐type transthyretin amyloidosis [26].

As expected, a length‐dependent, symmetrical axonal polyneuropathy was the most common type of non‐focal neuropathy found in our patients (sensory‐motor followed by sensory axonal polyneuropathy). This is similar to that observed in the four patients in the Italian cohort with the same variant who presented with polyneuropathy [15].

Notably, three of our patients with ATTR Val122Ile Amyloidosis presented with an atypical neuropathy pattern, including two patients with a sensorimotor multiple mononeuropathy with intermediate conduction velocity and one with a CIDP‐like pattern fulfilling the EAN/PNS 2021 criteria [19]. All these patients underwent extensive investigations to rule out other causes of neuropathy. Both patients with multiple mononeuropathy presented with associated cardiomyopathy, while the patient with the CIDP‐like pattern presented with neuropathy alone. The identification of atypical neuropathy presentations highlights the importance of maintaining a high index of suspicion for ATTRv amyloidosis even in patients with unusual presentations.

Although the typical pattern of amyloid neuropathy involves a diffuse, symmetrical, length‐dependent axonal disease [1], there are reports of asymmetric presentations, including patients with the Val122Ile variant [25, 27, 28, 29]. The CIDP‐like presentation has been cited as one of the main differential diagnoses of *TTR* amyloid neuropathy [30], although this presentation was quite uncommon in our series. A similar pattern has been found in patients with the Val30Met variant [27], and in a patient with the Val122Ile variant (who presented with multifocal mononeuropathies with features of demyelination on nerve conduction studies and a nerve biopsy demonstrating endoneural amyloid deposition) [29]. Therefore, *TTR* variants should always be considered in atypical or treatment‐unresponsive CIDP patients.

Radiculopathy was identified in some of our patients, but we are unsure of its significance. Some of them had spinal stenosis, based on clinical evaluation and imaging, which unfortunately was not performed in all centers. Furthermore, radiculopathy is a frequent finding in the general population, with a prevalence of lumbar radiculopathy estimated at 3%–5% of the population [31], and less frequent cervical radiculopathies at 1%–2% [32]. We suggest further studies for this purpose.

The SSR response was absent in 17.6% of the tested patients. Notably, it was never observed as an isolated finding. Although we have not studied this specifically, the association of CTS with the absence of SSR in a person without polyneuropathy may be suggestive of ATTR Val122Ile Amyloidosis, since SSR is typically preserved in CTS [33]. In patients with neuropathy, SSR was absent in just over half of the patients. A lower frequency of neurophysiological changes associated with small‐fiber involvement is expected in these patients, as small‐fiber neuropathy is not a frequent manifestation in these patients. This is in contrast to what is classically seen in other variants, especially the Val30Met variant, whose onset manifestations frequently involve small‐fiber involvement [1].

Although this is the largest study specifically dedicated to the Val122Ile, several limitations must be enumerated. It was a multicentric and retrospective study. The protocols used and the available instruments varied significantly among the centers. This was particularly relevant to the study of small nerve fibers, as many centers were not able to use sophisticated neurophysiological testing and skin biopsy. This limitation precluded the use of existing small‐fiber diagnostic criteria in most patients. As presented in the Methods section, we had to simplify the diagnostic criteria for small‐fiber neuropathy (thermoalgesic and autonomic manifestations with normal nerve conduction studies). Although multicentric, it only evaluated Brazilian patients. In other variants, including the Val30Met, presentations vary significantly according to the country. Lastly, there may have been a selection bias, since this study was carried out in neurology referral centers, and therefore a higher frequency of patients with neuropathy may have been found.

## 5 Conclusion

The Val122Ile variant was identified in White, Black and Pardo individuals. Neuropathic involvement was frequent in Val122Ile patients, traditionally considered to have a cardiac phenotype. Neuropathy is common at onset, being the only manifestation in 21.6% of patients after a five‐year follow‐up period. Sensory symptoms were the most common among patients with polyneuropathy. Dysautonomia, neuropathic pain, and motor impairment are less frequent than in ATTR Val30Met amyloidosis. The most common neuropathy pattern is an axonal length‐dependent symmetrical polyneuropathy, although atypical patterns (multiple mononeuropathy and CIDP‐like) were also observed.

These findings reinforce and expand the concept that TTR mutations should be considered in patients with neuropathy of unknown etiology, even with atypical phenotypes, and highlight the importance of routine neurological screening in asymptomatic patients carrying the Val122Ile variant. A greater understanding of the manifestations associated with *TTR* Val122Ile is critical for earlier diagnosis and treatment.

## Funding

This work was supported by Chamada CNPq/SECTICS/CAPES/FAPs No. 46/2024 (Process No. 409148/2024‐5).

## Conflicts of Interest

The authors declare no conflicts of interest.

## Supporting information


**Table S1:** Reason for excluding 120 individuals with the TTR Val122Ile variant from the general analysis of clinical and NCS/EMG findings for fulfilling one or more exclusion criteria.
**Figure S1:** Map with the slave route from Africa to Brazil showing the origin of Brazilian Blacks.

## Data Availability

The data that support the findings of this study are available on request from the corresponding author. The data are not publicly available due to privacy or ethical restrictions.
