# Supplementary material for: Neuropathy in Val122Ile Hereditary Transthyretin (ATTR) Amyloidosis: A Multicenter Retrospective Cohort Study
Source: J Peripher Nerv Syst. 2026 Aug 28;31(3):e70160. doi: 10.1111/jns.70160 (PMC13523732; doi:10.1111/jns.70160)
Supplement: Supplementary file 1 [file JNS-31-0-s001.docx]

**Supplemental material**

| **Exclusion criteria** | **Number of patients** |
| --- | --- |
| Compound heterozygous (Val122Ile/Ile107Val and Val122Ile/Val30Met) | 2 |
| Diabetes mellitus | 21 |
| Alcoholism | 6 |
| Leprosy | 1 |
| Possible Leprosy ^a^ | 1 |
| Neurotoxic drug use (Tacrolimus and Amiodarone) | 2 |
| Chronic renal failure of undefined cause | 1 |
| Motor neuron disease ^b^ | 2 |
| AA amyloidosis ^c^ | 1 |
| Charcot-Marie-Tooth disease ^d^ | 1 |
| Not having undergone an NCS/EMG | 90 |

***Supplementary Table 1.* Reason for excluding 120 individuals with the TTR Val122Ile variant from the general analysis of clinical and NCS/EMG findings for fulfilling one or more exclusion criteria.**

^a^ asymmetric neuropathy in which it was not possible to rule out leprosy due to death. ^b^ 1 patient with the classic form of motor neuron disease (upper and lower motor neuron involvement) and 1 patient with involvement only of the lower motor neuron associated with dysautonomia, but without findings of amyloid deposits in salivary gland, skin, nerve and muscle biopsies. ^c^ patient with neuropathy, renal failure, heart disease and hepatomegaly with liver biopsy that showed AA amyloid deposits. ^d^ patient with clinical features compatible with Charcot-Marie-Tooth disease and homozygous variant of the GDAP1 gene.

***Supplementary Figure 1.* Map with the slave route from Africa to Brazil showing the origin of Brazilian Blacks. (Adapted from Gomes)**^6^
